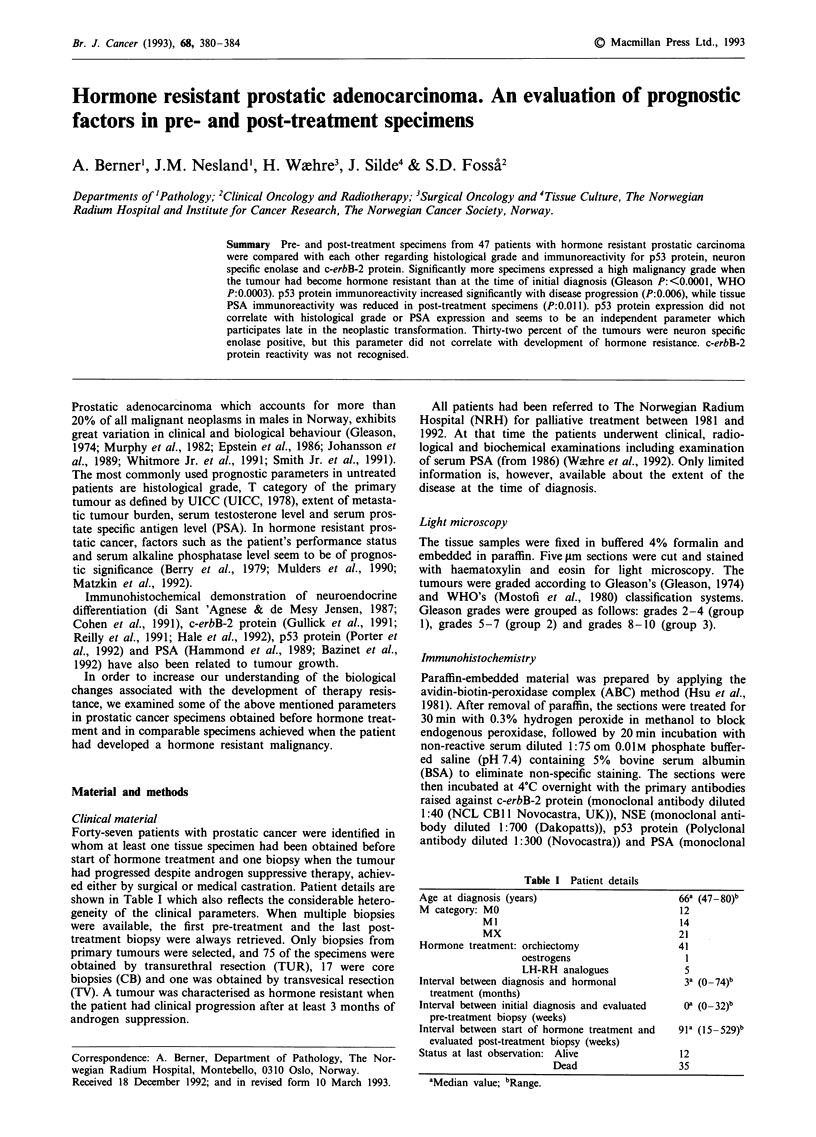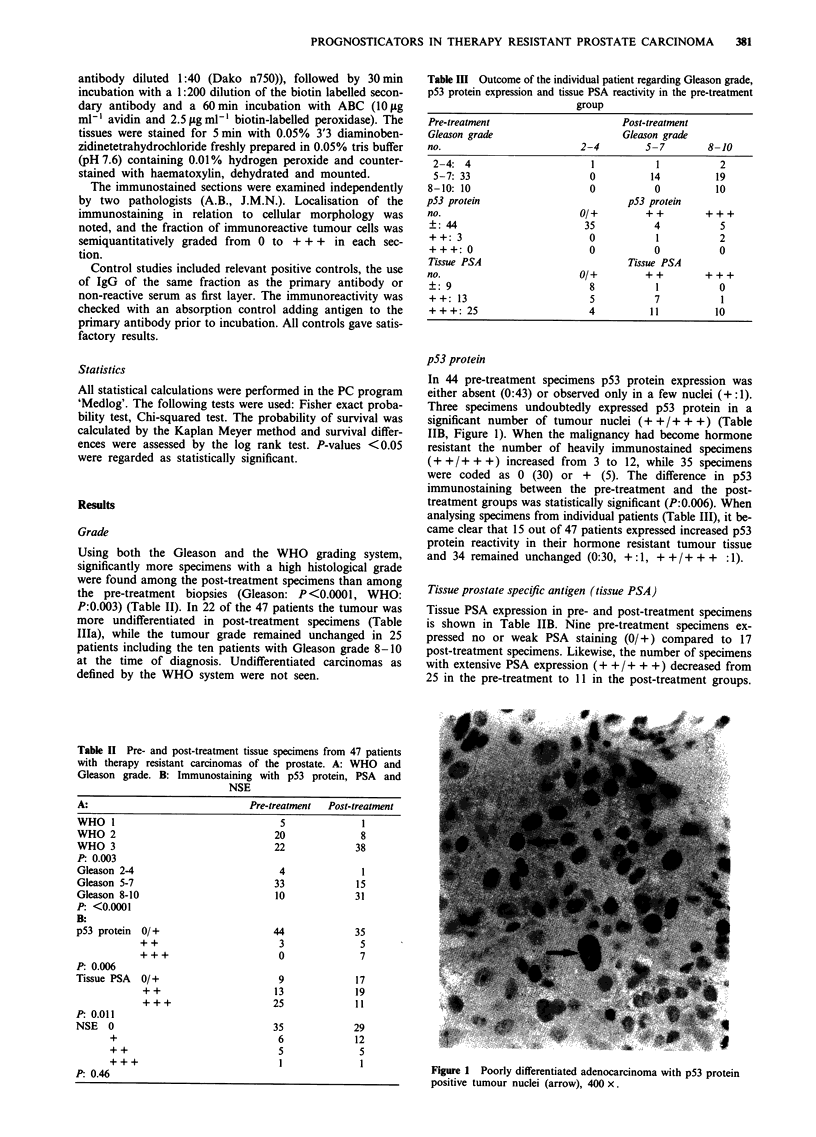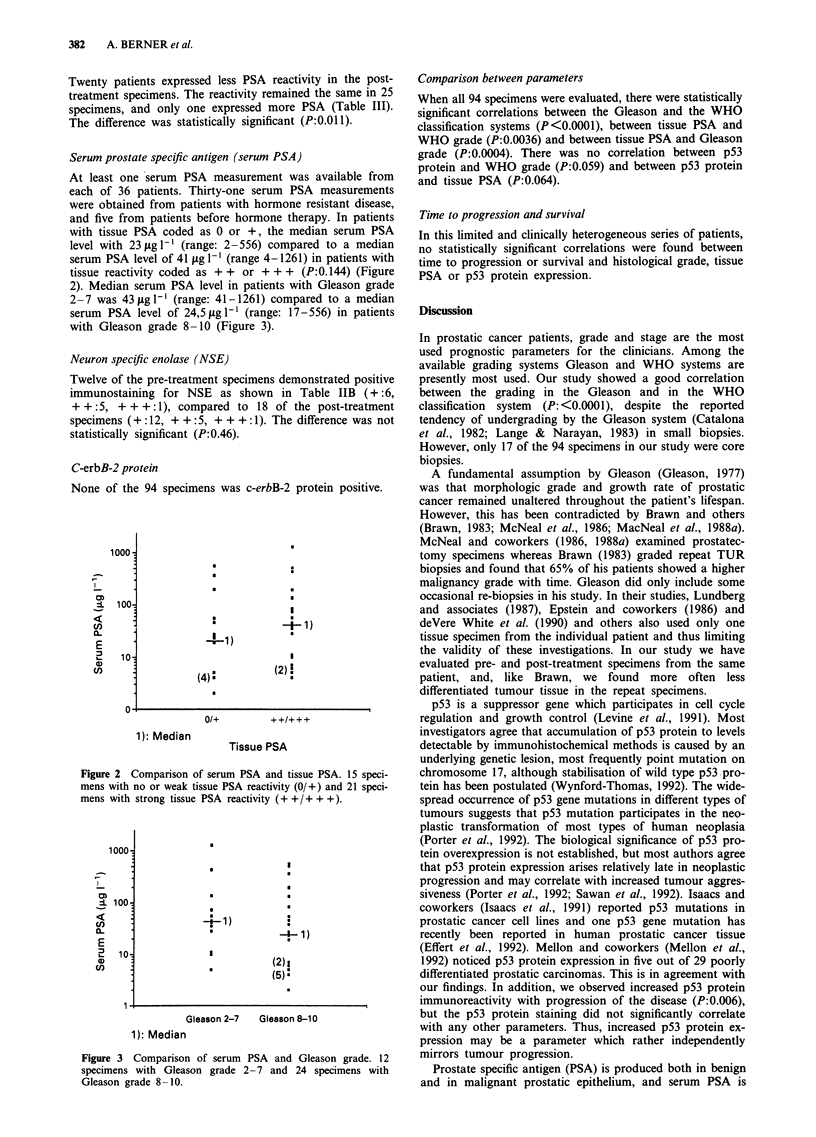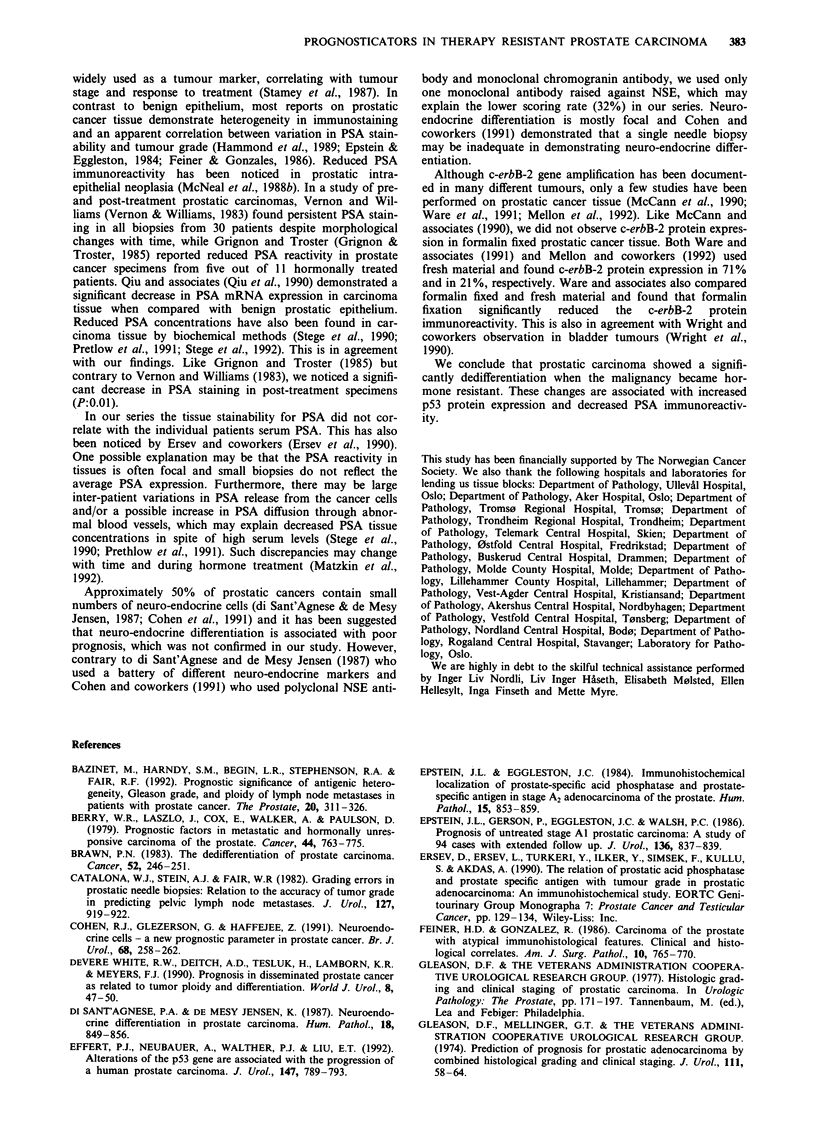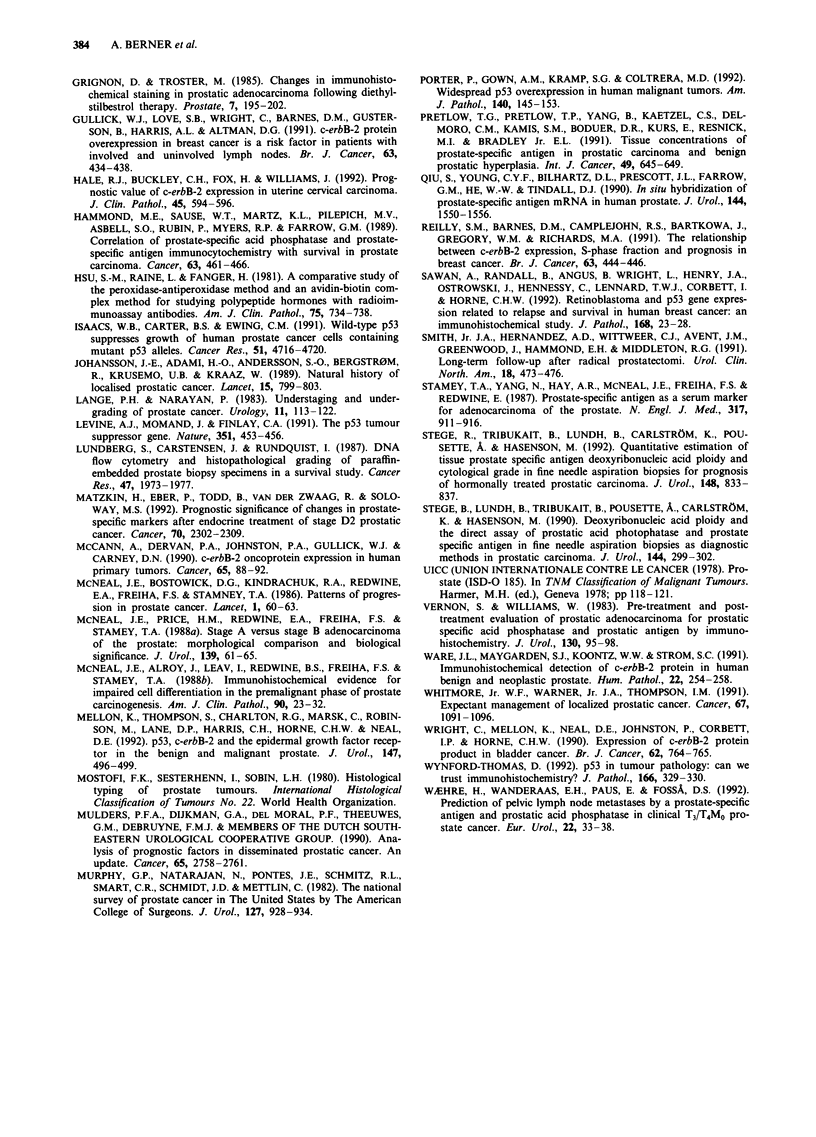# Hormone resistant prostatic adenocarcinoma. An evaluation of prognostic factors in pre- and post-treatment specimens.

**DOI:** 10.1038/bjc.1993.344

**Published:** 1993-08

**Authors:** A. Berner, J. M. Nesland, H. Waehre, J. Silde, S. D. Fosså

**Affiliations:** Department of Pathology, Norwegian Radium Hospital, Oslo.

## Abstract

**Images:**


Br. J. Cancer (1993), 68, 380-384 © Macmillan Press Ltd., 1993

### Hormone resistant prostatic adenocarcinoma. An evaluation of prognostic factors in pre- and post-treatment specimens

A. Berner[1], J.M. Nesland[1], H. Wæhre[3], J. Silde[4] & S.D. Fosså[2]

*Departments of [1]Pathology; [2]Clinical Oncology and Radiotherapy; [3]Surgical Oncology and [4]Tissue Culture, The Norwegian Radium Hospital and Institute for Cancer Research, The Norwegian Cancer Society, Norway.*

**Summary** Pre- and post-treatment specimens from 47 patients with hormone resistant prostatic carcinoma were compared with each other regarding histological grade and immunoreactivity for p53 protein, neuron specific enolase and c-*erb*B-2 protein. Significantly more specimens expressed a high malignancy grade when the tumour had become hormone resistant than at the time of initial diagnosis (Gleason $P$:<0.0001, WHO $P$:0.0003). p53 protein immunoreactivity increased significantly with disease progression ($P$:0.006), while tissue PSA immunoreactivity was reduced in post-treatment specimens ($P$:0.011). p53 protein expression did not correlate with histological grade or PSA expression and seems to be an independent parameter which participates late in the neoplastic transformation. Thirty-two percent of the tumours were neuron specific enolase positive, but this parameter did not correlate with development of hormone resistance. c-*erb*B-2 protein reactivity was not recognised.

Prostatic adenocarcinoma which accounts for more than 20% of all malignant neoplasms in males in Norway, exhibits great variation in clinical and biological behaviour (Gleason, 1974; Murphy *et al.*, 1982; Epstein *et al.*, 1986; Johansson *et al.*, 1989; Whitmore Jr. *et al.*, 1991; Smith Jr. *et al.*, 1991). The most commonly used prognostic parameters in untreated patients are histological grade, T category of the primary tumour as defined by UICC (UICC, 1978), extent of metastatic tumour burden, serum testosterone level and serum prostate specific antigen level (PSA). In hormone resistant prostatic cancer, factors such as the patient's performance status and serum alkaline phosphatase level seem to be of prognostic significance (Berry *et al.*, 1979; Mulders *et al.*, 1990; Matzkin *et al.*, 1992).

Immunohistochemical demonstration of neuroendocrine differentiation (di Sant 'Agnese & de Mesy Jensen, 1987; Cohen *et al.*, 1991), c-*erb*B-2 protein (Gullick *et al.*, 1991; Reilly *et al.*, 1991; Hale *et al.*, 1992), p53 protein (Porter *et al.*, 1992) and PSA (Hammond *et al.*, 1989; Bazinet *et al.*, 1992) have also been related to tumour growth.

In order to increase our understanding of the biological changes associated with the development of therapy resistance, we examined some of the above mentioned parameters in prostatic cancer specimens obtained before hormone treatment and in comparable specimens achieved when the patient had developed a hormone resistant malignancy.

### Material and methods

### Clinical material

Forty-seven patients with prostatic cancer were identified in whom at least one tissue specimen had been obtained before start of hormone treatment and one biopsy when the tumour had progressed despite androgen suppressive therapy, achieved either by surgical or medical castration. Patient details are shown in Table I which also reflects the considerable heterogeneity of the clinical parameters. When multiple biopsies were available, the first pre-treatment and the last post-treatment biopsy were always retrieved. Only biopsies from primary tumours were selected, and 75 of the specimens were obtained by transurethral resection (TUR), 17 were core biopsies (CB) and one was obtained by transvesical resection (TV). A tumour was characterised as hormone resistant when the patient had clinical progression after at least 3 months of androgen suppression.

Correspondence: A. Berner, Department of Pathology, The Norwegian Radium Hospital, Montebello, 0310 Oslo, Norway.
Received 18 December 1992; and in revised form 10 March 1993.

All patients had been referred to The Norwegian Radium Hospital (NRH) for palliative treatment between 1981 and 1992. At that time the patients underwent clinical, radiological and biochemical examinations including examination of serum PSA (from 1986) (Wæhre *et al.*, 1992). Only limited information is, however, available about the extent of the disease at the time of diagnosis.

### Light microscopy

The tissue samples were fixed in buffered 4% formalin and embedded in paraffin. Five µm sections were cut and stained with haematoxylin and eosin for light microscopy. The tumours were graded according to Gleason's (Gleason, 1974) and WHO's (Mostofi *et al.*, 1980) classification systems. Gleason grades were grouped as follows: grades 2-4 (group 1), grades 5-7 (group 2) and grades 8-10 (group 3).

### Immunohistochemistry

Paraffin-embedded material was prepared by applying the avidin-biotin-peroxidase complex (ABC) method (Hsu *et al.*, 1981). After removal of paraffin, the sections were treated for 30 min with 0.3% hydrogen peroxide in methanol to block endogenous peroxidase, followed by 20 min incubation with non-reactive serum diluted 1:75 om 0.01 M phosphate buffered saline (pH 7.4) containing 5% bovine serum albumin (BSA) to eliminate non-specific staining. The sections were then incubated at 4°C overnight with the primary antibodies raised against c-*erb*B-2 protein (monoclonal antibody diluted 1:40 (NCL CB11 Novocastra, UK)), NSE (monoclonal antibody diluted 1:700 (Dakopatts)), p53 protein (Polyclonal antibody diluted 1:300 (Novocastra)) and PSA (monoclonal

**Table I** Patient details

| | |
|---|---|
| Age at diagnosis (years) | 66[a] (47-80)[b] |
| M category: M0 | 12 |
| M1 | 14 |
| MX | 21 |
| Hormone treatment: orchiectomy | 41 |
| oestrogens | 1 |
| LH-RH analogues | 5 |
| Interval between diagnosis and hormonal treatment (months) | 3[a] (0-74)[b] |
| Interval between initial diagnosis and evaluated pre-treatment biopsy (weeks) | 0[a] (0-32)[b] |
| Interval between start of hormone treatment and evaluated post-treatment biopsy (weeks) | 91[a] (15-529)[b] |
| Status at last observation: Alive | 12 |
| Dead | 35 |

[a]Median value; [b]Range.

antibody diluted 1:40 (Dako n750)), followed by 30 min incubation with a 1:200 dilution of the biotin labelled secondary antibody and a 60 min incubation with ABC (10 µg $ml^{-1}$ avidin and 2.5 µg $ml^{-1}$ biotin-labelled peroxidase). The tissues were stained for 5 min with 0.05% 3'3 diaminobenzidinetetrahydrochloride freshly prepared in 0.05% tris buffer (pH 7.6) containing 0.01% hydrogen peroxide and counterstained with haematoxylin, dehydrated and mounted.

The immunostained sections were examined independently by two pathologists (A.B., J.M.N.). Localisation of the immunostaining in relation to cellular morphology was noted, and the fraction of immunoreactive tumour cells was semiquantitatively graded from 0 to + + + in each section.

Control studies included relevant positive controls, the use of IgG of the same fraction as the primary antibody or non-reactive serum as first layer. The immunoreactivity was checked with an absorption control adding antigen to the primary antibody prior to incubation. All controls gave satisfactory results.

*Statistics*

All statistical calculations were performed in the PC program 'Medlog'. The following tests were used: Fisher exact probability test, Chi-squared test. The probability of survival was calculated by the Kaplan Meyer method and survival differences were assessed by the log rank test. *P*-values <0.05 were regarded as statistically significant.

### Results

*Grade*

Using both the Gleason and the WHO grading system, significantly more specimens with a high histological grade were found among the post-treatment specimens than among the pre-treatment biopsies (Gleason: *P*<0.0001, WHO: *P*:0.003) (Table II). In 22 of the 47 patients the tumour was more undifferentiated in post-treatment specimens (Table IIIa), while the tumour grade remained unchanged in 25 patients including the ten patients with Gleason grade 8–10 at the time of diagnosis. Undifferentiated carcinomas as defined by the WHO system were not seen.

**Table II** Pre- and post-treatment tissue specimens from 47 patients with therapy resistant carcinomas of the prostate. **A**: WHO and Gleason grade. **B**: Immunostaining with p53 protein, PSA and NSE

| A: | | *Pre-treatment* | *Post-treatment* |
|---|---|---|---|
| WHO 1 | | 5 | 1 |
| WHO 2 | | 20 | 8 |
| WHO 3 | | 22 | 38 |
| *P*: 0.003 | | | |
| Gleason 2-4 | | 4 | 1 |
| Gleason 5-7 | | 33 | 15 |
| Gleason 8-10 | | 10 | 31 |
| *P*: <0.0001 | | | |
| **B:** | | | |
| p53 protein | 0/+ | 44 | 35 |
| | + + | 3 | 5 |
| | + + + | 0 | 7 |
| *P*: 0.006 | | | |
| Tissue PSA | 0/+ | 9 | 17 |
| | + + | 13 | 19 |
| | + + + | 25 | 11 |
| *P*: 0.011 | | | |
| NSE | 0 | 35 | 29 |
| | + | 6 | 12 |
| | + + | 5 | 5 |
| | + + + | 1 | 1 |
| *P*: 0.46 | | | |

**Table III** Outcome of the individual patient regarding Gleason grade, p53 protein expression and tissue PSA reactivity in the pre-treatment group

| *Pre-treatment Gleason grade no.* | *2–4* | *Post-treatment Gleason grade 5–7* | *8–10* |
|---|---|---|---|
| 2–4: 4 | 1 | 1 | 2 |
| 5–7: 33 | 0 | 14 | 19 |
| 8–10: 10 | 0 | 0 | 10 |
| *p53 protein no.* | *0/+* | *p53 protein + +* | *+ + +* |
| ±: 44 | 35 | 4 | 5 |
| + +: 3 | 0 | 1 | 2 |
| + + +: 0 | 0 | 0 | 0 |
| *Tissue PSA no.* | *0/+* | *Tissue PSA + +* | *+ + +* |
| ±: 9 | 8 | 1 | 0 |
| + +: 13 | 5 | 7 | 1 |
| + + +: 25 | 4 | 11 | 10 |

*p53 protein*

In 44 pre-treatment specimens p53 protein expression was either absent (0:43) or observed only in a few nuclei (+:1). Three specimens undoubtedly expressed p53 protein in a significant number of tumour nuclei (+ +/+ + +) (Table IIB, Figure 1). When the malignancy had become hormone resistant the number of heavily immunostained specimens (+ +/+ + +) increased from 3 to 12, while 35 specimens were coded as 0 (30) or + (5). The difference in p53 immunostaining between the pre-treatment and the post-treatment groups was statistically significant (*P*:0.006). When analysing specimens from individual patients (Table III), it became clear that 15 out of 47 patients expressed increased p53 protein reactivity in their hormone resistant tumour tissue and 34 remained unchanged (0:30, +:1, + +/+ + + :1).

*Tissue prostate specific antigen (tissue PSA)*

Tissue PSA expression in pre- and post-treatment specimens is shown in Table IIB. Nine pre-treatment specimens expressed no or weak PSA staining (0/+) compared to 17 post-treatment specimens. Likewise, the number of specimens with extensive PSA expression (+ +/+ + +) decreased from 25 in the pre-treatment to 11 in the post-treatment groups.

**Figure 1** Poorly differentiated adenocarcinoma with p53 protein positive tumour nuclei (arrow), 400 ×.

Twenty patients expressed less PSA reactivity in the post-treatment specimens. The reactivity remained the same in 25 specimens, and only one expressed more PSA (Table III). The difference was statistically significant (*P*:0.011).

*Serum prostate specific antigen (serum PSA)*

At least one serum PSA measurement was available from each of 36 patients. Thirty-one serum PSA measurements were obtained from patients with hormone resistant disease, and five from patients before hormone therapy. In patients with tissue PSA coded as 0 or +, the median serum PSA level with 23 $\mu g\,l^{-1}$ (range: 2–556) compared to a median serum PSA level of 41 $\mu g\,l^{-1}$ (range 4–1261) in patients with tissue reactivity coded as + + or + + + (*P*:0.144) (Figure 2). Median serum PSA level in patients with Gleason grade 2–7 was 43 $\mu g\,l^{-1}$ (range: 41–1261) compared to a median serum PSA level of 24,5 $\mu g\,l^{-1}$ (range: 17–556) in patients with Gleason grade 8–10 (Figure 3).

*Neuron specific enolase (NSE)*

Twelve of the pre-treatment specimens demonstrated positive immunostaining for NSE as shown in Table IIB (+:6, + +:5, + + +:1), compared to 18 of the post-treatment specimens (+:12, + +:5, + + +:1). The difference was not statistically significant (*P*:0.46).

*C*-erb*B-2 protein*

None of the 94 specimens was c-*erb*B-2 protein positive.

**Figure 2** Comparison of serum PSA and tissue PSA. 15 specimens with no or weak tissue PSA reactivity (0/+) and 21 specimens with strong tissue PSA reactivity (+ +/+ + +).

**Figure 3** Comparison of serum PSA and Gleason grade. 12 specimens with Gleason grade 2–7 and 24 specimens with Gleason grade 8–10.

*Comparison between parameters*

When all 94 specimens were evaluated, there were statistically significant correlations between the Gleason and the WHO classification systems ($P<0.0001$), between tissue PSA and WHO grade (*P*:0.0036) and between tissue PSA and Gleason grade (*P*:0.0004). There was no correlation between p53 protein and WHO grade (*P*:0.059) and between p53 protein and tissue PSA (*P*:0.064).

*Time to progression and survival*

In this limited and clinically heterogeneous series of patients, no statistically significant correlations were found between time to progression or survival and histological grade, tissue PSA or p53 protein expression.

**Discussion**

In prostatic cancer patients, grade and stage are the most used prognostic parameters for the clinicians. Among the available grading systems Gleason and WHO systems are presently most used. Our study showed a good correlation between the grading in the Gleason and in the WHO classification system (*P*:<0.0001), despite the reported tendency of undergrading by the Gleason system (Catalona *et al.*, 1982; Lange & Narayan, 1983) in small biopsies. However, only 17 of the 94 specimens in our study were core biopsies.

A fundamental assumption by Gleason (Gleason, 1977) was that morphologic grade and growth rate of prostatic cancer remained unaltered throughout the patient's lifespan. However, this has been contradicted by Brawn and others (Brawn, 1983; McNeal *et al.*, 1986; MacNeal *et al.*, 1988*a*). McNeal and coworkers (1986, 1988*a*) examined prostatectomy specimens whereas Brawn (1983) graded repeat TUR biopsies and found that 65% of his patients showed a higher malignancy grade with time. Gleason did only include some occasional re-biopsies in his study. In their studies, Lundberg and associates (1987), Epstein and coworkers (1986) and deVere White *et al.* (1990) and others also used only one tissue specimen from the individual patient and thus limiting the validity of these investigations. In our study we have evaluated pre- and post-treatment specimens from the same patient, and, like Brawn, we found more often less differentiated tumour tissue in the repeat specimens.

p53 is a suppressor gene which participates in cell cycle regulation and growth control (Levine *et al.*, 1991). Most investigators agree that accumulation of p53 protein to levels detectable by immunohistochemical methods is caused by an underlying genetic lesion, most frequently point mutation on chromosome 17, although stabilisation of wild type p53 protein has been postulated (Wynford-Thomas, 1992). The widespread occurrence of p53 gene mutations in different types of tumours suggests that p53 mutation participates in the neoplastic transformation of most types of human neoplasia (Porter *et al.*, 1992). The biological significance of p53 protein overexpression is not established, but most authors agree that p53 protein expression arises relatively late in neoplastic progression and may correlate with increased tumour aggressiveness (Porter *et al.*, 1992; Sawan *et al.*, 1992). Isaacs and coworkers (Isaacs *et al.*, 1991) reported p53 mutations in prostatic cancer cell lines and one p53 gene mutation has recently been reported in human prostatic cancer tissue (Effert *et al.*, 1992). Mellon and coworkers (Mellon *et al.*, 1992) noticed p53 protein expression in five out of 29 poorly differentiated prostatic carcinomas. This is in agreement with our findings. In addition, we observed increased p53 protein immunoreactivity with progression of the disease (*P*:0.006), but the p53 protein staining did not significantly correlate with any other parameters. Thus, increased p53 protein expression may be a parameter which rather independently mirrors tumour progression.

Prostate specific antigen (PSA) is produced both in benign and in malignant prostatic epithelium, and serum PSA is widely used as a tumour marker, correlating with tumour stage and response to treatment (Stamey *et al.*, 1987). In contrast to benign epithelium, most reports on prostatic cancer tissue demonstrate heterogeneity in immunostaining and an apparent correlation between variation in PSA stainability and tumour grade (Hammond *et al.*, 1989; Epstein & Eggleston, 1984; Feiner & Gonzales, 1986). Reduced PSA immunoreactivity has been noticed in prostatic intraepithelial neoplasia (McNeal *et al.*, 1988*b*). In a study of pre- and post-treatment prostatic carcinomas, Vernon and Williams (Vernon & Williams, 1983) found persistent PSA staining in all biopsies from 30 patients despite morphological changes with time, while Grignon and Troster (Grignon & Troster, 1985) reported reduced PSA reactivity in prostate cancer specimens from five out of 11 hormonally treated patients. Qiu and associates (Qiu *et al.*, 1990) demonstrated a significant decrease in PSA mRNA expression in carcinoma tissue when compared with benign prostatic epithelium. Reduced PSA concentrations have also been found in carcinoma tissue by biochemical methods (Stege *et al.*, 1990; Pretlow *et al.*, 1991; Stege *et al.*, 1992). This is in agreement with our findings. Like Grignon and Troster (1985) but contrary to Vernon and Williams (1983), we noticed a significant decrease in PSA staining in post-treatment specimens ($P$:0.01).

In our series the tissue stainability for PSA did not correlate with the individual patients serum PSA. This has also been noticed by Ersev and coworkers (Ersev *et al.*, 1990). One possible explanation may be that the PSA reactivity in tissues is often focal and small biopsies do not reflect the average PSA expression. Furthermore, there may be large inter-patient variations in PSA release from the cancer cells and/or a possible increase in PSA diffusion through abnormal blood vessels, which may explain decreased PSA tissue concentrations in spite of high serum levels (Stege *et al.*, 1990; Prethlow *et al.*, 1991). Such discrepancies may change with time and during hormone treatment (Matzkin *et al.*, 1992).

Approximately 50% of prostatic cancers contain small numbers of neuro-endocrine cells (di Sant'Agnese & de Mesy Jensen, 1987; Cohen *et al.*, 1991) and it has been suggested that neuro-endocrine differentiation is associated with poor prognosis, which was not confirmed in our study. However, contrary to di Sant'Agnese and de Mesy Jensen (1987) who used a battery of different neuro-endocrine markers and Cohen and coworkers (1991) who used polyclonal NSE antibody and monoclonal chromogranin antibody, we used only one monoclonal antibody raised against NSE, which may explain the lower scoring rate (32%) in our series. Neuro-endocrine differentiation is mostly focal and Cohen and coworkers (1991) demonstrated that a single needle biopsy may be inadequate in demonstrating neuro-endocrine differentiation.

Although c-*erb*B-2 gene amplification has been documented in many different tumours, only a few studies have been performed on prostatic cancer tissue (McCann *et al.*, 1990; Ware *et al.*, 1991; Mellon *et al.*, 1992). Like McCann and associates (1990), we did not observe c-*erb*B-2 protein expression in formalin fixed prostatic cancer tissue. Both Ware and associates (1991) and Mellon and coworkers (1992) used fresh material and found c-*erb*B-2 protein expression in 71% and in 21%, respectively. Ware and associates also compared formalin fixed and fresh material and found that formalin fixation significantly reduced the c-*erb*B-2 protein immunoreactivity. This is also in agreement with Wright and coworkers observation in bladder tumours (Wright *et al.*, 1990).

We conclude that prostatic carcinoma showed a significantly dedifferentiation when the malignancy became hormone resistant. These changes are associated with increased p53 protein expression and decreased PSA immunoreactivity.

This study has been financially supported by The Norwegian Cancer Society. We also thank the following hospitals and laboratories for lending us tissue blocks: Department of Pathology, Ullevål Hospital, Oslo; Department of Pathology, Aker Hospital, Oslo; Department of Pathology, Tromsø Regional Hospital, Tromsø; Department of Pathology, Trondheim Regional Hospital, Trondheim; Department of Pathology, Telemark Central Hospital, Skien; Department of Pathology, Østfold Central Hospital, Fredrikstad; Department of Pathology, Buskerud Central Hospital, Drammen; Department of Pathology, Molde County Hospital, Molde; Department of Pathology, Lillehammer County Hospital, Lillehammer; Department of Pathology, Vest-Agder Central Hospital, Kristiansand; Department of Pathology, Akershus Central Hospital, Nordbyhagen; Department of Pathology, Vestfold Central Hospital, Tønsberg; Department of Pathology, Nordland Central Hospital, Bodø; Department of Pathology, Rogaland Central Hospital, Stavanger; Laboratory for Pathology, Oslo.

We are highly in debt to the skilful technical assistance performed by Inger Liv Nordli, Liv Inger Håseth, Elisabeth Mølsted, Ellen Hellesylt, Inga Finseth and Mette Myre.

### References

BAZINET, M., HARNDY, S.M., BEGIN, L.R., STEPHENSON, R.A. & FAIR, R.F. (1992). Prognostic significance of antigenic heterogeneity, Gleason grade, and ploidy of lymph node metastases in patients with prostate cancer. *The Prostate*, **20**, 311–326.

BERRY, W.R., LASZLO, J., COX, E., WALKER, A. & PAULSON, D. (1979). Prognostic factors in metastatic and hormonally unresponsive carcinoma of the prostate. *Cancer*, **44**, 763–775.

BRAWN, P.N. (1983). The dedifferentiation of prostate carcinoma. *Cancer*, **52**, 246–251.

CATALONA, W.J., STEIN, A.J. & FAIR, W.R (1982). Grading errors in prostatic needle biopsies: Relation to the accuracy of tumor grade in predicting pelvic lymph node metastases. *J. Urol.*, **127**, 919–922.

COHEN, R.J., GLEZERSON, G. & HAFFEJEE, Z. (1991). Neuroendocrine cells – a new prognostic parameter in prostate cancer. *Br. J. Urol.*, **68**, 258–262.

DEVERE WHITE, R.W., DEITCH, A.D., TESLUK, H., LAMBORN, K.R. & MEYERS, F.J. (1990). Prognosis in disseminated prostate cancer as related to tumor ploidy and differentiation. *World J. Urol.*, **8**, 47–50.

DI SANT'AGNESE, P.A. & DE MESY JENSEN, K. (1987). Neuroendocrine differentiation in prostate carcinoma. *Hum. Pathol.*, **18**, 849–856.

EFFERT, P.J., NEUBAUER, A., WALTHER, P.J. & LIU, E.T. (1992). Alterations of the p53 gene are associated with the progression of a human prostate carcinoma. *J. Urol.*, **147**, 789–793.

EPSTEIN, J.L. & EGGLESTON, J.C. (1984). Immunohistochemical localization of prostate-specific acid phosphatase and prostate-specific antigen in stage $A_2$ adenocarcinoma of the prostate. *Hum. Pathol.*, **15**, 853–859.

EPSTEIN, J.L., GERSON, P., EGGLESTON, J.C. & WALSH, P.C. (1986). Prognosis of untreated stage A1 prostatic carcinoma: A study of 94 cases with extended follow up. *J. Urol.*, **136**, 837–839.

ERSEV, D., ERSEV, L., TURKERI, Y., ILKER, Y., SIMSEK, F., KULLU, S. & AKDAS, A. (1990). The relation of prostatic acid phosphatase and prostate specific antigen with tumour grade in prostatic adenocarcinoma: An immunohistochemical study. EORTC Genitourinary Group Monographa 7: *Prostate Cancer and Testicular Cancer*, pp. 129–134, Wiley-Liss: Inc.

FEINER, H.D. & GONZALEZ, R. (1986). Carcinoma of the prostate with atypical immunohistological features. Clinical and histological correlates. *Am. J. Surg. Pathol.*, **10**, 765–770.

GLEASON, D.F. & THE VETERANS ADMINISTRATION COOPERATIVE UROLOGICAL RESEARCH GROUP. (1977). Histologic grading and clinical staging of prostatic carcinoma. In *Urologic Pathology: The Prostate*, pp. 171–197. Tannenbaum, M. (ed.), Lea and Febiger: Philadelphia.

GLEASON, D.F., MELLINGER, G.T. & THE VETERANS ADMINISTRATION COOPERATIVE UROLOGICAL RESEARCH GROUP. (1974). Prediction of prognosis for prostatic adenocarcinoma by combined histological grading and clinical staging. *J. Urol.*, **111**, 58–64.

GRIGNON, D. & TROSTER, M. (1985). Changes in immunohistochemical staining in prostatic adenocarcinoma following diethylstilbestrol therapy. *Prostate*, **7**, 195–202.

GULLICK, W.J., LOVE, S.B., WRIGHT, C., BARNES, D.M., GUSTERSON, B., HARRIS, A.L. & ALTMAN, D.G. (1991). c-*erb*B-2 protein overexpression in breast cancer is a risk factor in patients with involved and uninvolved lymph nodes. *Br. J. Cancer*, **63**, 434–438.

HALE, R.J., BUCKLEY, C.H., FOX, H. & WILLIAMS, J. (1992). Prognostic value of c-*erb*B-2 expression in uterine cervical carcinoma. *J. Clin. Pathol.*, **45**, 594–596.

HAMMOND, M.E., SAUSE, W.T., MARTZ, K.L., PILEPICH, M.V., ASBELL, S.O., RUBIN, P., MYERS, R.P. & FARROW, G.M. (1989). Correlation of prostate-specific acid phosphatase and prostate-specific antigen immunocytochemistry with survival in prostate carcinoma. *Cancer*, **63**, 461–466.

HSU, S.-M., RAINE, L. & FANGER, H. (1981). A comparative study of the peroxidase-antiperoxidase method and an avidin-biotin complex method for studying polypeptide hormones with radioimmunoassay antibodies. *Am. J. Clin. Pathol.*, **75**, 734–738.

ISAACS, W.B., CARTER, B.S. & EWING, C.M. (1991). Wild-type p53 suppresses growth of human prostate cancer cells containing mutant p53 alleles. *Cancer Res.*, **51**, 4716–4720.

JOHANSSON, J.-E., ADAMI, H.-O., ANDERSSON, S.-O., BERGSTRØM, R., KRUSEMO, U.B. & KRAAZ, W. (1989). Natural history of localised prostatic cancer. *Lancet*, **15**, 799–803.

LANGE, P.H. & NARAYAN, P. (1983). Understaging and undergrading of prostate cancer. *Urology*, **11**, 113–122.

LEVINE, A.J., MOMAND, J. & FINLAY, C.A. (1991). The p53 tumour suppressor gene. *Nature*, **351**, 453–456.

LUNDBERG, S., CARSTENSEN, J. & RUNDQUIST, I. (1987). DNA flow cytometry and histopathological grading of paraffin-embedded prostate biopsy specimens in a survival study. *Cancer Res.*, **47**, 1973–1977.

MATZKIN, H., EBER, P., TODD, B., VAN DER ZWAAG, R. & SOLOWAY, M.S. (1992). Prognostic significance of changes in prostate-specific markers after endocrine treatment of stage D2 prostatic cancer. *Cancer*, **70**, 2302–2309.

MCCANN, A., DERVAN, P.A., JOHNSTON, P.A., GULLICK, W.J. & CARNEY, D.N. (1990). c-*erb*B-2 oncoprotein expression in human primary tumors. *Cancer*, **65**, 88–92.

MCNEAL, J.E., BOSTOWICK, D.G., KINDRACHUK, R.A., REDWINE, E.A., FREIHA, F.S. & STAMNEY, T.A. (1986). Patterns of progression in prostate cancer. *Lancet*, **1**, 60–63.

MCNEAL, J.E., PRICE, H.M., REDWINE, E.A., FREIHA, F.S. & STAMEY, T.A. (1988*a*). Stage A versus stage B adenocarcinoma of the prostate: morphological comparison and biological significance. *J. Urol.*, **139**, 61–65.

MCNEAL, J.E., ALROY, J., LEAV, I., REDWINE, B.S., FREIHA, F.S. & STAMEY, T.A. (1988*b*). Immunohistochemical evidence for impaired cell differentiation in the premalignant phase of prostate carcinogenesis. *Am. J. Clin. Pathol.*, **90**, 23–32.

MELLON, K., THOMPSON, S., CHARLTON, R.G., MARSK, C., ROBINSON, M., LANE, D.P., HARRIS, C.H., HORNE, C.H.W. & NEAL, D.E. (1992). p53, c-*erb*B-2 and the epidermal growth factor receptor in the benign and malignant prostate. *J. Urol.*, **147**, 496–499.

MOSTOFI, F.K., SESTERHENN, I., SOBIN, L.H. (1980). Histological typing of prostate tumours. *International Histological Classification of Tumours No. 22*. World Health Organization.

MULDERS, P.F.A., DIJKMAN, G.A., DEL MORAL, P.F., THEEUWES, G.M., DEBRUYNE, F.M.J. & MEMBERS OF THE DUTCH SOUTH-EASTERN UROLOGICAL COOPERATIVE GROUP. (1990). Analysis of prognostic factors in disseminated prostatic cancer. An update. *Cancer*, **65**, 2758–2761.

MURPHY, G.P., NATARAJAN, N., PONTES, J.E., SCHMITZ, R.L., SMART, C.R., SCHMIDT, J.D. & METTLIN, C. (1982). The national survey of prostate cancer in The United States by The American College of Surgeons. *J. Urol.*, **127**, 928–934.

PORTER, P., GOWN, A.M., KRAMP, S.G. & COLTRERA, M.D. (1992). Widespread p53 overexpression in human malignant tumors. *Am. J. Pathol.*, **140**, 145–153.

PRETLOW, T.G., PRETLOW, T.P., YANG, B., KAETZEL, C.S., DELMORO, C.M., KAMIS, S.M., BODUER, D.R., KURS, E., RESNICK, M.I. & BRADLEY Jr. E.L. (1991). Tissue concentrations of prostate-specific antigen in prostatic carcinoma and benign prostatic hyperplasia. *Int. J. Cancer*, **49**, 645–649.

QIU, S., YOUNG, C.Y.F., BILHARTZ, D.L., PRESCOTT, J.L., FARROW, G.M., HE, W.-W. & TINDALL, D.J. (1990). *In situ* hybridization of prostate-specific antigen mRNA in human prostate. *J. Urol.*, **144**, 1550–1556.

REILLY, S.M., BARNES, D.M., CAMPLEJOHN, R.S., BARTKOWA, J., GREGORY, W.M. & RICHARDS, M.A. (1991). The relationship between c-*erb*B-2 expression, S-phase fraction and prognosis in breast cancer. *Br. J. Cancer*, **63**, 444–446.

SAWAN, A., RANDALL, B., ANGUS, B. WRIGHT, L., HENRY, J.A., OSTROWSKI, J., HENNESSY, C., LENNARD, T.W.J., CORBETT, I. & HORNE, C.H.W. (1992). Retinoblastoma and p53 gene expression related to relapse and survival in human breast cancer: an immunohistochemical study. *J. Pathol.*, **168**, 23–28.

SMITH, Jr. J.A., HERNANDEZ, A.D., WITTWEER, C.J., AVENT, J.M., GREENWOOD, J., HAMMOND, E.H. & MIDDLETON, R.G. (1991). Long-term follow-up after radical prostatectomi. *Urol. Clin. North. Am.*, **18**, 473–476.

STAMEY, T.A., YANG, N., HAY, A.R., MCNEAL, J.E., FREIHA, F.S. & REDWINE, E. (1987). Prostate-specific antigen as a serum marker for adenocarcinoma of the prostate. *N. Engl. J. Med.*, **317**, 911–916.

STEGE, R., TRIBUKAIT, B., LUNDH, B., CARLSTRÖM, K., POUSETTE, Å. & HASENSON, M. (1992). Quantitative estimation of tissue prostate specific antigen deoxyribonucleic acid ploidy and cytological grade in fine needle aspiration biopsies for prognosis of hormonally treated prostatic carcinoma. *J. Urol.*, **148**, 833–837.

STEGE, B., LUNDH, B., TRIBUKAIT, B., POUSETTE, Å., CARLSTRÖM, K. & HASENSON, M. (1990). Deoxyribonucleic acid ploidy and the direct assay of prostatic acid photophatase and prostate specific antigen in fine needle aspiration biopsies as diagnostic methods in prostatic carcinoma. *J. Urol.*, **144**, 299–302.

UICC (UNION INTERNATIONALE CONTRE LE CANCER (1978). Prostate (ISD-O 185). In *TNM Classification of Malignant Tumours*. Harmer, M.H. (ed.), Geneva 1978; pp 118–121.

VERNON, S. & WILLIAMS, W. (1983). Pre-treatment and post-treatment evaluation of prostatic adenocarcinoma for prostatic specific acid phosphatase and prostatic antigen by immunohistochemistry. *J. Urol.*, **130**, 95–98.

WARE, J.L., MAYGARDEN, S.J., KOONTZ, W.W. & STROM, S.C. (1991). Immunohistochemical detection of c-*erb*B-2 protein in human benign and neoplastic prostate. *Hum. Pathol.*, **22**, 254–258.

WHITMORE, Jr. W.F., WARNER, Jr. J.A., THOMPSON, I.M. (1991). Expectant management of localized prostatic cancer. *Cancer*, **67**, 1091–1096.

WRIGHT, C., MELLON, K., NEAL, D.E., JOHNSTON, P., CORBETT, I.P. & HORNE, C.H.W. (1990). Expression of c-*erb*B-2 protein product in bladder cancer. *Br. J. Cancer*, **62**, 764–765.

WYNFORD-THOMAS, D. (1992). p53 in tumour pathology: can we trust immunohistochemistry? *J. Pathol.*, **166**, 329–330.

WÆHRE, H., WANDERAAS, E.H., PAUS, E. & FOSSÅ, D.S. (1992). Prediction of pelvic lymph node metastases by a prostate-specific antigen and prostatic acid phosphatase in clinical $T_3/T_4M_0$ prostate cancer. *Eur. Urol.*, **22**, 33–38.